# The Role of the Microbiome in Gastroentero-Pancreatic Neuroendocrine Neoplasms (GEP-NENs)

**DOI:** 10.3390/cimb44050136

**Published:** 2022-04-30

**Authors:** Amr Mohamed, Sylvia L. Asa, Thomas McCormick, Hilmi Al-Shakhshir, Arvind Dasari, Retuerto Mauricio, Iman Salem, Lee M. Ocuin, David Bajor, Richard T. Lee, J. Eva Selfridge, Arash Kardan, Zhenghong Lee, Norbert Avril, Shelby Kopp, Jordan M. Winter, Jeffrey M. Hardacre, John B. Ammori, Mahmoud A. Ghannoum

**Affiliations:** 1 Division of Hematology and Medical Oncology, UH Seidman Cancer Center, University Hospitals Cleveland Medical Center, Cleveland, OH 44106, USA; david.bajor@uhhospitals.org (D.B.); rtl24@case.edu (R.T.L.); jennifer.selfridge@uhhospitals.org (J.E.S.); shelby.kopp@uhhospitals.org (S.K.); 2 Department of Pathology, UH Seidman Cancer Center, Case Comprehensive Cancer Center, Case Western Reserve University, Cleveland, OH 44106, USA; sylvia.asa@uhhospitals.org; 3 Department of Dermatology, Integrated Microbiome Core and Center for Medical Mycology, Case Western Reserve University, Cleveland, OH 44106, USA; thomas.mccormick@uhhospitals.org (T.M.); hra44@case.edu (H.A.-S.); mar153@case.edu (R.M.); iman.salem@uhhospitals.org (I.S.); mag3@case.edu (M.A.G.); 4 Department of Gastrointestinal Medical Oncology, MD Anderson Cancer Center, University of Texas, Houston, TX 77030, USA; adasari@mdanderson.org; 5 Division of Surgical Oncology, UH Seidman Cancer Center, University Hospitals Cleveland Medical Center, Cleveland, OH 44106, USA; lee.ocuin@uhhospitals.org (L.M.O.); jordan.winter@uhhospitals.org (J.M.W.); jeffrey.hardacre@uhhospitals.org (J.M.H.); john.ammori@uhhospitals.org (J.B.A.); 6 Department of Radiology, UH Seidman Cancer Center, University Hospitals Cleveland Medical Center, Cleveland, OH 44106, USA; arash.kardan@uhhospitals.org (A.K.); zxl11@case.edu (Z.L.); norbert.avril@uhhospitals.org (N.A.)

**Keywords:** microbiome, neuroendocrine tumors

## Abstract

Gut microbiome balance plays a key role in human health and maintains gut barrier integrity. Dysbiosis, referring to impaired gut microbiome, is linked to a variety of diseases, including cancers, through modulation of the inflammatory process. Most studies concentrated on adenocarcinoma of different sites with very limited information on gastroenteropancreatic neuroendocrine neoplasms (GEP-NENs). In this study, we have analyzed the gut microbiome (both fungal and bacterial communities) in patients with metastatic GEP-NENs. Fecal samples were collected and compared with matched healthy control samples using logistic regression distances utilizing R package MatchIt (version 4.2.0, Daniel E. Ho, Stanford, CA, USA). We examined differences in microbiome profiles between GEP-NENs and control samples using small subunit (SSU) rRNA (16S), ITS1, ITS4 genomic regions for their ability to accurately characterize bacterial and fungal communities. We correlated the results with different behavioral and dietary habits, and tumor features including differentiation, grade, primary site, and therapeutic response. All tests are two-sided and *p*-values ≤ 0.05 were considered statistically significant. Gut samples of 34 patients (12 males, 22 females, median age 64 years) with metastatic GEP-NENs (22 small bowel, 10 pancreatic, 1 gall bladder, and 1 unknown primary) were analyzed. Twenty-nine patients had well differentiated GEP-neuroendocrine tumors (GEP-NETs), (G1 = 14, G2 = 12, G3 = 3) and five patients had poorly differentiated GEP-neuroendocrine carcinomas (GEP-NECs). Patients with GEP-NENs had significantly decreased bacterial species and increased fungi (notably *Candida* species, Ascomycota, and species belonging to saccharomycetes) compared to controls. Patients with GEP-NECs had significantly enriched populations of specific bacteria and fungi (such as *Enterobacter hormaechei*, *Bacteroides fragilis* and *Trichosporon asahii*) compared to those with GEP-NETs (*p* = 0.048, 0.0022 and 0.034, respectively). In addition, higher grade GEP-NETs were associated with significantly higher *Bacteroides fragilis* (*p* = 0.022), and *Eggerthella lenta* (*p* = 0.00018) species compared to lower grade tumors. There were substantial differences associated with dietary habits and therapeutic responses. This is the first study to analyze the role of the microbiome environment in patients with GEP-NENs. There were significant differences between GEP-NETs and GEP-NECs, supporting the role of the gut microbiome in the pathogenesis of these two distinct entities.

## 1. Introduction

Neuroendocrine neoplasms (NENs) are a diverse group of neoplasms that arise from endocrine cells throughout the body; a significant proportion originate in the gastrointestinal tract [1]. A Surveillance, Epidemiology, and End Results (SEER) analysis showed seven-fold increase in the incidence of gastroenteropancreatic neuroendocrine neoplasms (GEP-NENs) over the last decade in the United States [2]. NENs are classified according to histological differentiation, grade, tumor burden, origin of the primary tumor, and extent of metastatic disease. Tumor classification based on differentiation, referring to the extent to which neoplastic cells morphologically resemble endocrine cells of origin, divides GEP-NENs into well-differentiated neuroendocrine tumors (NETs) and poorly differentiated neuroendocrine carcinomas (NECs) [3]. NETs are graded based on proliferation as determined by mitotic activity and/or Ki67 labeling; by definition, NECs are all high grade. This classification has prognostic and therapeutic implications in which lower grade well-differentiated tumors tend to have an indolent course with a good outcome, while high-grade well-differentiated (grade 3) NETs and NECs tend to be aggressive malignancies with associated poor prognosis [3,4].

The gut microbiome, composed of diverse communities of bacteria, fungi, viruses, archaea, and protozoa, plays a key role in several metabolic functions and influences host immunity and general health [5]. The co-evolution of microbes and man has occurred over millions of years, and the inter-relationships between microbiome balance and the human body have undoubtedly shaped our health in the background of genetic mutations and immune modulation [6]. Thus, understanding the interrelationships of bacterial/fungal communities in the human microbiome is a significant challenge in the context of several human diseases, including cancers. Dysbiosis is defined as an imbalance in microbiome diversity; it is not limited to an exclusive imbalance in the bacterial community (Bacteriome, BM), but also involves alterations in the fungal community (Mycobiome, MYC). Several studies have highlighted that changes in the intestinal microbiome may have a major role in the development, differentiation [7,8], and progression of several cancers [9]. These studies demonstrated that an imbalance of the gut microbiome confers a predisposition to certain diseases and malignancies, and it could significantly influence therapeutic responses [10]. Additionally, recent data clarify the role of microbiome in host immunity and how it could significantly influence response and toxicity to immunotherapy [11]. While several studies have focused on the association between microbiome diversity and numerous gastrointestinal cancers, very little is known about the impact of the microbiome in GEP-NEN tumorigenesis. In this study, we analyzed the microbiome profile in patients with different GEP-NEN subgroups and evaluated their association with tumor differentiation and behavior.

## 2. Materials and Methods

### 2.1. Participants

All participants were recruited at the University Hospitals Seidman Cancer Center, Cleveland, Ohio. Written informed consent was obtained from all participants in this study before collection of the samples. Recruitment of study participants was performed according to protocol (#20200733) approved by the Human Subjects Institution Review Board (IRB) of Case Western Reserve University, Cleveland (OH), and UH Cleveland Medical Center, Cleveland (OH). We collected fecal samples from 40 GEP-NEN patients and 40 age- and sex-matched healthy controls. The healthy control cohort consisted of subjects who had screening colonoscopies and were exposed to the same environment and share similar dietary habits. Both patients and healthy subjects were American inhabitants and most of them consumed a western diet. Race was classified as White (including European, Hispanic, and East Indian), Black/African American, Asian, and Native American/Native Alaskan, etc. Current dietary habits, intake of antibiotics in the last 6 months before enrollment, intake of probiotics or vitamins, smoking status, alcohol intake in addition to age, gender, and ethnicity were all collected from GEP-NEN participants. Summary of demographic information of subjects enrolled in the study are shown in Table 1.

### 2.2. Sample Collection and Processing

Fecal samples were collected using a specific kit consisting of a ready-to-use package (BD BBL^TM^ CultureSwab), including a user guide. The samples were immediately placed in previously prepared fast prep tubes (MP, Cat# 5076-200-34340) containing 500 μL glass beads (Sigma-Aldrich G8772-100 g, St. Louis, MI, USA) and 1 mL ASL™ lysis buffer (Qiagen DNA Extraction Kit) and were transported to the laboratory where assays were conducted. Samples were kept in a −20 °C freezer until they were analyzed.

### 2.3. DNA Extraction

The QIA amp Fast DNA Stool Mini kit (Qiagen GmpH, Hilden, Germany) was used to extract DNA via the manufacturer’s instructions. Samples were pelleted by centrifugation and re-suspended in 1 mL of InhibitEX lysis Buffer. Fecal swabs were incubated for 1 h at 75 °C and mixed in Fastprep 96 2× for 300 s at 1800 RPM. Equal volumes of 100% ethanol and lysate were mixed and passed over HiBind DNA Mini Columns (Omega Bio-tek, Norcross, GA, USA). The DNA extract was eluted into 50 µL of molecular grade water. The quality and purity of the isolated genomic DNA was assessed by gel electrophoresis and quantified using a Qubit 2.0 instrument and Qubit dsDNA HS Assay (Life Technologies, Carlsbad, CA, USA). DNA samples were stored at −20 °C.

### 2.4. PCR Amplification

Amplification of the 16S and 5.8S rRNA genes was performed using 16S-515 (5′-GGA CTA CCA GGG TAT CTA ATC CTG-3′) and 16S -804 (5′-(TCC TAC GGG AGG CAG CAG T-3′) and ITS1 (5′-(TCC GTA GGT GAA CCT GCG G-3′) and ITS4 (5′-TCC TCC GCT TAT TGA TAT GC-3′) primers, respectively [12,13]. PCR products were prepared in Q5 High-Fidelity Master Mix (New England Bioinformatics) (1 × concentration), together with a double volume of molecular grade water and 0.05 µL/mM of each primer. Undiluted source DNA (1.5 μL) was then added to each 50 µL PCR reaction. Thermo-cycling conditions used an initial denaturation step of 3 min at 98 °C, followed by 30 cycles of denaturation at for 10 s at 98 °C, annealing for 10 s at 55 °C for the 16S primers and 20 s at 58 °C for the ITS primers, and an extension step of 10 s at 72 °C followed by a final extension step of 3 min at 72 °C. PCR products were separated by gel electrophoresis on a 1.5% agarose gel containing 7 μg/mL ethidium bromide.

### 2.5. Library Preparation and Sequencing

Amplicon libraries were cleaned and barcoded followed by emulsion PCR using Ion Torrent S5 Prime workflow (Thermo-Fisher, Waltham, MA, USA). Bacterial 16S rRNA and fungal ITS amplicons of equal volumes were pooled, cleaned using AMPure XP beads (Beckman Coulter, Brea, CA, USA) and then exposed to end repair enzyme for 20 min at RT. Following an additional AMPure clean up, ligation reactions were performed at 25 °C for 30 min using an Ion Torrent P1 with a unique barcoded ′A′ adaptor per pooled sample. AMPure removal of residual adaptors was performed, and samples were concentrated to 1/4 volume for 1 h under heat and vacuum. Separate barcoded samples were pooled in equal amounts (10 μL) and size selected for the anticipated 16S and ITS range (200–800 bp) using Pippin Prep (Sage Bioscience, Beverly, MA, USA). Library amplification was performed for seven cycles and quantified using a StepOne qPCR instrument prior to proper dilution to 300 pM before adding the samples into IonSphere templating reactions (Ion Chef). Library sequencing was performed using an Ion Torrent S5 sequencer (ThermoFisher Scientific). Barcoded and sorted samples were analyzed using our custom pipeline based on Greengenes V13_8 and UNITE database V7.2 designed for the taxonomic classification of 16SrRNA and ITS sequences, respectively. Sequencing reads were clustered into operational taxonomic units (OTUs, 3% distance), described by community metrics, and classified within the Qiime 1.8 taxonomy bioinformatics pipeline.

### 2.6. Data Preparation

Raw 16s and ITS data of the bacterial read counts was obtained and was loaded to R version 4.0.3., sample meta data was obtained, and, using R package microbiome 1.12.0 and phyloseq 1.34.0 a phyloseq object containing a read count matrix (species level identification), a taxonomic table (kingdom to species) and metadata table were made. When dealing with both 16s and ITS data, each is initially handled separately and then combined after the read counts are normalized and transformed to Relative abundance.

### 2.7. Pre-Processing and Quality Control (QC)

Prior to creating the phyloseq object for the 16s and ITS data, the taxonomic table is cleaned to remove species and phyla that are annotated as “unidentified” or lacking identification at a species level annotated as an empty cell. This step allows us to ensure only species that are identifiable are being utilized in the analysis.

The main filtering step performed is on the total read counts per sample, where a cutoff of 500 read counts in the 16s data is used as a minimum requirement. This brings down the total number of samples from 40 to 34 samples. Additionally, samples with missing metadata annotations were also removed.

### 2.8. Data Exploration

Composition bar graphs were generated on 16s and ITS relative abundance data separately after aggregating the data to the phyla level, and filtering on phyla prevalence of 25% amongst all samples. This allows for an overview of differences in the core Phyla between samples.

Ordinate analysis/PCA was performed on microbiome and mycobiome data separately, after aggregating data to the phyla level as a means to reduce complexity. The data are then filtered on prevalence of 75% amongst all samples. Ordinate analysis is performed using the “ordinate()” function part of microbiome R package version 1.12.0 with method used as “MDS” (multidimensional scaling) and distances using “Bray–Curtis” method.

Venn diagrams were generated using the eulerr R package version 6.1.0, using the 16s and ITS species level relative abundance. The core microbiome within each group is determined using a prevalence of 50% within samples in each group.

### 2.9. Data Analysis

16s and ITS relative abundance data are first filtered on species with a prevalence of 50% within all samples, and then the respective phyloseq objects are merged. Wilcoxon-rank-sum nonparametric test is used to test for significantly different species between the groups being compared, and the fold change is calculated using mean relative abundance of a species within each group using the fold change function from R package tools version 3.9.2. For clinical features that contained more than 2 groups, a Kruskal–Wallis test is used to test for significance in difference between groups. Additionally, Spearman correlation is used to test for significant correlation between clinical features and species within samples. Box plots using ggplot2 R package were generated for all the significantly different species.

## 3. Results

We studied the stool microbiota of 34 patients with GEP-NENs, as well as bacterial and fungal communities of 34 matched healthy individuals. Initially, 40 samples were collected, but 6 were not included due to either insufficient fecal sample collection or failed QC. Thus, samples from 34 patients (12 males, 22 females, median age 64 years) were included in the final analysis. All participants were from the metropolitan Cleveland, Ohio area. All had metastatic GEP-NENs (22 small bowel, 10 pancreatic, 1 gall bladder, and 1 unknown primary); 29 patients were GEP-NETs, (G1 = 14, G2 = 12, G3 = 3) and 5 were GEP-NECs.

**Figure 1:**
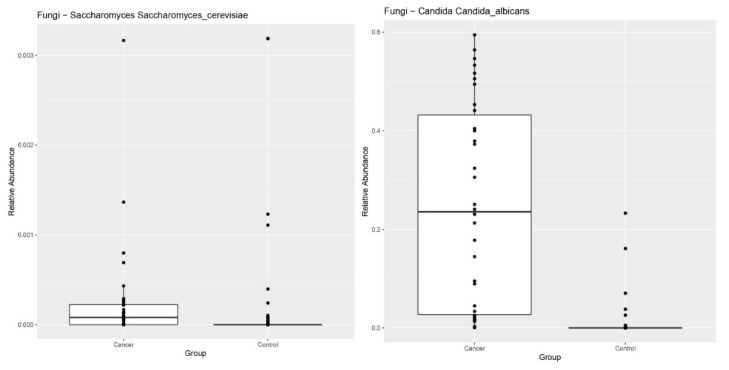
Patients with GEP-NENs had significantly decreased relative abundance of bacterial species and an increase in the relative abundance of fungi (*Candida* species, Ascomycota, and species belonging to saccharomycetes) compared to controls (*p* = 0.0013).

Comparison of the microbiome profiles of patients with GEP-NENs and healthy controls showed a significant reduction in the relative abundance of bacterial species and an increase in the relative abundance of fungi, notably *Candida*, Ascomycota, and species belonging to saccharomycetes, (*p* = 0.0013), compared to controls (Figure 1). Although other fungal phyla were present in fecal samples of GEP-NEN patients, they were not statistically significant compared to healthy controls.

**Figure 2:**
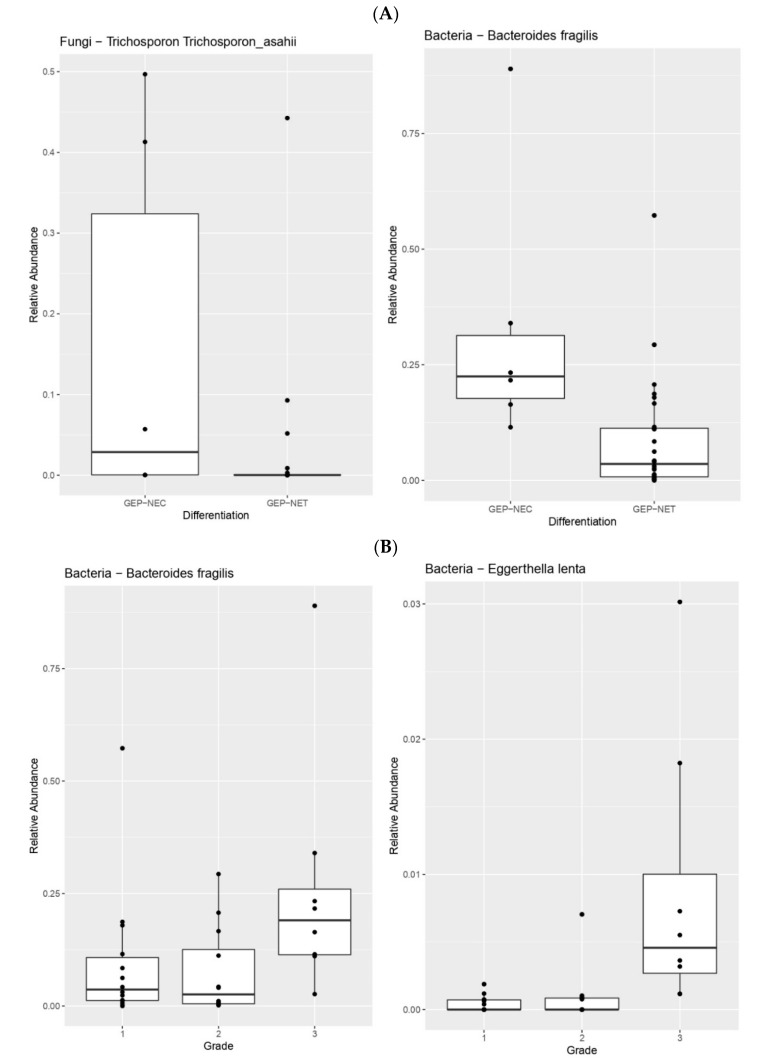
(**A**). Patients with GEP-NECs have significantly enriched bacteria and fungi (*Enterobacter hormaechei*, *Bacteroides fragilis*, and *Trichosporon asahii*) compared with patients who have GEP-NETs (*p* = 0.048, 0.0022, and 0.034 respectively). (**B**). Higher grade (G3) GEP-NETs were associated with significantly higher *Bacteroides fragilis* (*p* = 0.022) and *Eggerthella lenta* (*p* = 0.00018) species compared to lower grade GEP-NETs (G1/2).

When we compared the microbiome profile between different GEP-NEN subgroups according to their histological differentiation and grades, the results demonstrated significant differences. Patients with GEP-NECs had significantly enriched bacteria and fungi, especially *Enterobacter hormaechei*, *Bacteroides fragilis*, and *Trichosporon asahii*, compared to those with GEP-NETs (*p* = 0.048, 0.0028 and 0.046, respectively), (Figure 2A). In addition, higher grade (G3) GEP-NETs were associated with significantly higher *Bacteroides fragilis* (*p* = 0.031), *Eggerthella lenta (p =* 0.00028), and Bacteroidetes Prevotella (*p* = 0.032) species compared to lower grade (G1 & G2) GEP-NETs (Figure 2B).

**Figure 3:**
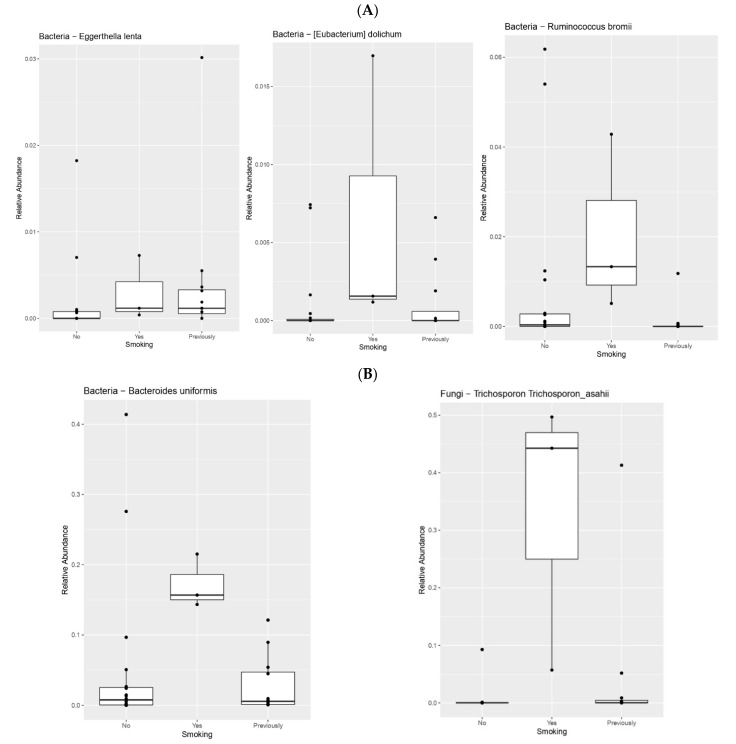
(**A**) Smokers had significantly more anaerobic, Gram-positive species (*Eggerthella lenta*, *Eubacterium dolichum*, and *Ruminococcus bromii*) (*p* = 0.03, 0.046, and 0.01, respectively) compared to non-smokers. (**B**) Smokers had significantly more anaerobic Gram-negative bacteria (*Bacteroides uniformis*) (*p* = 0.04), and certain fungi (*Trichosporon asahii*) (*p* = 0.005) compared to non-smokers.

There were significant differences in the microbiome profile associated with smoking, alcohol intake, and antibiotics between patients with GEP-NENs. Smokers had significantly more anaerobic, Gram-positive species, such as *Eggerthella lenta*, *Eubacterium dolichum*, and *Ruminococcus bromii* (*p* = 0.03, 0.046, and 0.01, respectively); anaerobic, Gram-negative *Bacteroides uniformis* (*p* = 0.04); and certain fungi (*Trichosporon asahii*), (*p* = 0.005) compared to non-smokers (Figure 3) (Table 2).

**Figure 4:**
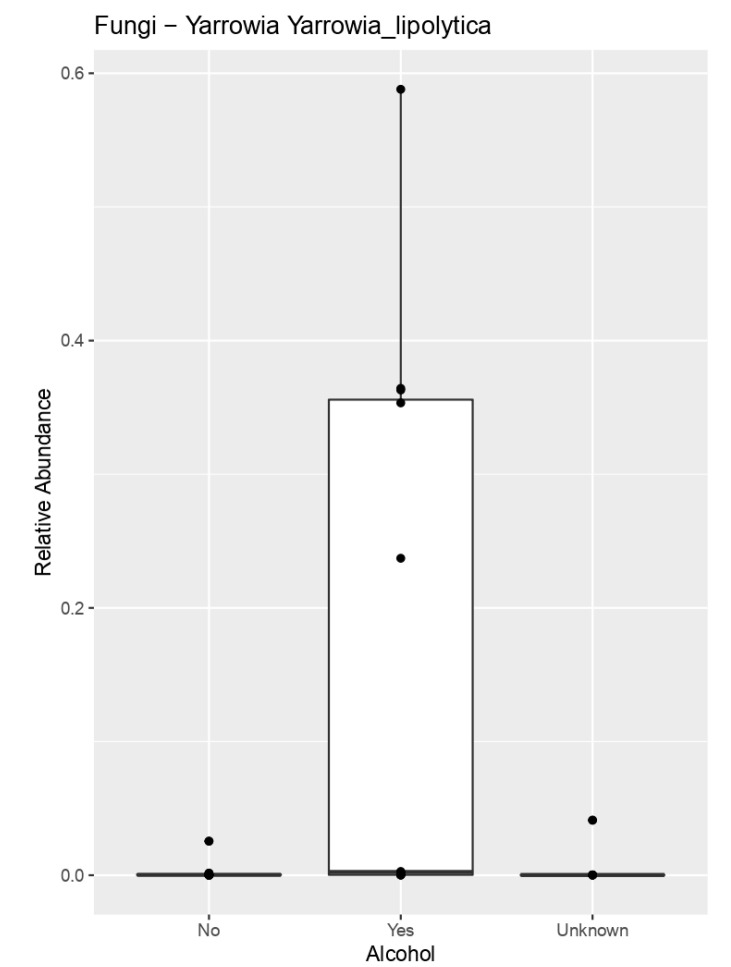
Patients who drink alcohol regularly (more than two times per week) had significantly more fungal species (*Yarrowia lipolytica*, *p* = 0.022) compared to those who denied alcohol intake.

When we compared alcohol intake between GEP-NEN patients, patients that drink alcohol regularly (more than two times per week) had significantly more fungal species such as (*Yarrowia lipolytica*, *p* = 0.022) and certain bacterial species such as the Gram-positive non-spore-forming bacilli (*Collinsella aerofaciens*, *p* = 0.03) compared to those who deny alcohol consumption (Figure 4).

Additionally, we analyzed the microbiome profile of GEP-NEN patients who regularly consumed probiotics or had taken antibiotics in the 6 months before sample collection. The results indicated that patients who took antibiotics in the last 6 months had relatively more abundance of certain fungal species (*Candida albicans, Candida dubliniensis, Candida tropicalis*, and *Trichosporon mucoides*; *p* = 0.0074, 0.0049, 0.0054, and 0.019, respectively) compared to patients who did not receive antibiotics. Patients who ingest probiotics regularly had more abundance of certain bacterial (*Bacteroidetes Prevotella*, *Lactobacillus zeae*, and *Anoxybacillus kestanbolensis*; *p* = 0.0189, 0.0133, and 0.047, respectively), and fungal species (*Metarhizium anisopliae* and *Trichosporon mucoides*; *p* = 0.0024 and 0.0097, respectively).

We compared the microbiome profile according to the dietary habits to identify the relationship between habitual dietary intake and microbiome variation. Patients who consumed vegetables at least five times a week had certain bacterial and fungal species (*Faecalibacterium prausnitzii and Ruminococcus bromii*; *p =* 0.08 and 0.066, respectively) which were different from those who consumed mainly an animal protein based diet (*Bacteroides eggerthii, Staphylococcus epidermidis*, and *Citrobacter gillenii*; *p =* 0.065, 0.07, and 0.03, respectively). Patients with a high carbohydrate diet and those who consumed sweets, on average, six times per week had higher bacterial species, such as *Bifidobacterium Adolescentis, Blautia producta*, and *Agrobacterium (p =* 0.056, 0.060, and 0.078, respectively) [14,15] (Table 2).

## 4. Discussion

There is evolving evidence supporting the role of the gut microbiome in the pathogenesis of several diseases, including cancers [16,17]. Impaired gut microbiota, called dysbiosis, results from an imbalance in the microbiome equilibrium and can be linked with different tumors. Most of the previous studies concentrated on several gastrointestinal cancers, including hepatocellular carcinoma (HCC), and colon and pancreatic cancers with very limited information on NENs [18,19,20]. Our study is the first to analyze the complex interplay between the gut microbiome and GEP-NENs and raises the possibility that the microbiome may play a role in the development and progression of these tumors.

We found significant differences in the bacterial (bacteriome) and fungal (mycobiome) species between patients with GEP-NENs and healthy subjects, with an increase in the relative abundance of fungi, especially *Candida, Ascomycota*, and *Saccharomycetes* species. Currently there is a growing interest in the associations between the human mycobiome and its potential role in carcinogenesis. Previous studies have shown that mycobiome diversity and density are usually low in healthy subjects [21]. Certain factors, such as proton pump inhibitors (PPIs) or antibiotic-induced dysbiosis of intestinal microbes, can predispose individuals to *Candida albicans* colonization and this has been linked to the oncogenic process in many cancers including pancreatic ductal adenocarcinoma (PDA), colorectal carcinoma (CRC), and head and neck carcinoma (HNCC) [22]. There are several mechanisms by which fungal dysbiosis can participate in the etiopathogenesis of cancers. *Candida albicans* can produce cytolytic toxic peptides called candidalysin and acetaldehyde, which are known to disrupt the epithelial barrier function and increase intracellular reactive oxygen species (ROS) that lead to mitochondrial dysfunction and cytoxicity [23,24]. Thus, the mycobiome may be implicated in carcinogenesis and could represent a novel therapeutic target in the future.

Our results indicate that there are significant bacteriome differences between histological subgroups of GEP-NENs. Patients with high-grade, well differentiated GEP-NETs and poorly differentiated GEP-NECs had significantly enriched *Bacteroides fragilis* (*B. fragilis*) species. Previous studies have shown that *B. fragilis* colonization in the intestinal tract and production of *B. fragilis* toxin (*BFT*) can disrupt the intestinal environment and lead to chronic inflammation and tissue injury, which has correlated with tumorigenesis including colorectal cancer [25,26]. *B. fragilis* and the *BFT* gene were found to be significantly higher in colorectal cancer patients compared to healthy controls [25]. It has been proposed that *BFT* exposure in the human colon may induce chronic IL-17–dependent inflammation, causing epithelial barrier damage and oxidative DNA damage, resulting in increased risk of colorectal carcinoma [25,26,27]. The same study highlighted the association between *B. Fragilis* and advanced colorectal cancer stages given that the presence of *BFT* gene in patients with stage III cancer was significantly higher than in patients with stages I and II cancers [25]. These results confirmed the findings of Boleij et al. and Viljoen et al. studies, in which *BFT* was detected particularly in the late stages of colorectal carcinoma patients [28,29]. Therefore, the correlation between *B. fragilis* with high-grade NETs and NECs need further exploration.

Additionally, patients with poorly differentiated GEP-NECs had significantly enriched bacteria and fungi (Especially *Enterobacter hormaechei* and *T. asahii*) compared to those with GEP-NETs. Several previous studies have been performed to determine the interaction between Enterobacter species and cancer. Dilşad Yurdakul et al. investigated the impact of Enterobacter protein on colon cancer cell lines [30]. The results showed that proteins isolated from the Enterobacter species significantly increased cell viability and proliferation while decreasing apoptosis, indicating that Enterobacter strains might promote colon cancer [30]. *T. asahii* species are non-*Candida* fungi found widely in nature, and are related to superficial and opportunistic invasive infections in cancer patients. Studies have shown that *T. asahii* is a part of the dysbiosis signature of mycobiota in several cancers [31]. These studies suggest that alterations in the surrounding microenvironment of tumors by gut mycobiome, including *T. asahii*, alter the host immune system and correlate with the development and progression of colorectal cancer [31,32].

Our results indicated that higher grade (G3) GEP-NETs were also associated with significantly higher *Eubacterium dolichum* and *Eggerthella lenta* species, in addition to *B. Fragilis* compared to lower grade tumors. *E. dolichum* is a Gram-positive bacterium in the family Eubacteriaceae, while *E. lenta* is an anaerobic Gram-positive bacillus. Both are considered pro-inflammatory bacteria and have been linked to certain cancers, such as breast and colon cancer [33,34]. The information about the role of these members of the bacteriome and mycobiome in cancer aggressiveness and differentiation is still limited and needs further investigation.

Previous studies have highlighted the role of the microbiome in cancers, however there is no specific cause–effect relationship that has been conclusively demonstrated. Several mechanisms have been proposed concerning how dysbiosis induces carcinogenesis. These include chronic inflammation triggering cell transformation, stimulation of pro-inflammatory cytokines (IL-6, IL-8, and TNF), alteration of the tumor microenvironment, and production of reactive oxygen species (ROS) and nitric oxygen synthase (NOS) that can lead to DNA damage [35,36]. For example, overproduction of reactive oxygen and nitrogen species by *Bacteroides fragilis* toxin and *Colibactin* expressed by *Escherichia coli* can cause indirect DNA damage, and are associated with colorectal carcinogenesis [37,38]. The only data available for NENs concern the role of *Helicobacter Pylori (HP)* infection that has been shown to activate the PI3K-AKT-mTOR pathway, which plays a relevant role in the pathogenesis and progression of gastric NENs [39]. Interestingly, HP infection induces PTEN phosphorylation, which activates AKT and inhibits apoptosis. In addition, HP stimulate vascular endothelial growth factor receptor expression in human microvascular endothelial cells (HMEC- 1) which induces angiogenesis that plays a vital role in highly vascular cancers including NENs [40]. Therefore, the impact of the microbiome and mycobiome on the PI3K-AKT-mTOR pathway and angiogenesis in GEP-NENs warrant further investigations.

Our study has some limitations including small sample size, and its retrospective nature. Other limitations include the lack of investigating the impact of therapies on microbiome for enrolled patients; furthermore, there was a lack of uniformity with regards to the therapy received by patients. Despite these limitations, this is the first retrospective study investigating the role of microbiome in patients with different GEP-NENs differentiation and grades.

## 5. Conclusions

We provide the first analysis of the bacteriome and mycobiome in patients with GEP-NENs. The results show significant differences between histological subgroups of GEP-NENs. Further studies are needed to validate these results in larger numbers of patients and to determine the impact of the various hormones they produce, as well as the effect of the various therapies, including somatostatin analogues, on the bacteriome and mycobiome. These studies should concentrate on the potential role of bacteria, fungi, and viruses in the development and progression of GEP-NENs and how they may impact therapeutic responses. Understanding the relationships between different microbiome species and GEP-NEN tumorigenesis will shed light on the pathogenicity of these organisms and may lead to the discovery of novel therapeutic approaches, which could have relevant clinical implications in modifying therapy of these tumors.

## Figures and Tables

**Table 1:** Summary of Demographics.

Characteristics	Neuroendocrine Neoplasms	Healthy Controls
Numbers	34	34
Median Age	64 (57–70)	67 (56–74)
Male %	35%	41%
Primary site	22 small bowel	None
	10 pancreatic	
	1 gallbladder	
	1 unknown primary	
Histological Differentiation		NA
NET	29	
G1	14	
G2	12	
G3	3	
NEC	5	
Dietary habits	Number of patients	
Red meat/Times per week		
0	4	
1	15	
2	11	
3	2	
4	2	
Chicken/Times per week		
0	1	
1	8	
2	8	
3	8	
4	6	
5	2	
6	1	
Fish/Times per week		
0	2	
1	22	
2	6	
3	1	
5	1	
7	1	
Vegetables/Times per week		
2	1	
3	2	
4	2	
5	2	
7	25	
14	2	
Sweets/Times per week		
0	2	
1	4	
2	9	
3	4	
4	1	
5	1	
6	1	
7	12	
Antibiotic Use in last 6 months		NA
Yes	9	
No	25	

**Table 2:** Microbiome species associated with different dietary habits, antibiotic exposure and therapeutic responses.

	Microbiome Diversity	*p*-Value/Rho
Smoking	*Eggerthella lenta*	0.03171159609
	*Bacteroides uniformis*	0.04712580917
	*Parabacteroides distasonis*	0.02032498682
	*Ruminococcus bromii*	0.01317982135
	*Eubacterium dolichum*	0.04625801676
	*Shigella flexneri*	0.03093423221
	*Trichosporon asahii*	0.005986598077
Alcohol	*Collinsella aerofaciens*	0.0370489641
	*Yarrowia lipolytica*	0.02290117753
	*Trichosporon asahii*	0.03762051
Antibiotic Use	*Candida albicans*	0.007496000617
	*Candida dubliniensis*	0.004912107598
	*Candida Tropicalis*	0.005475873728
	*Candida* sp.	0.008415458738
	*Trichosporon mucoides*	0.01966306396
Probiotic Use	*Bacteroidetes Prevotella*	0.01892686135
	*Lactobacillus zeae*	0.01330485808
	*Anoxybacillus kestanbolensis*	0.04702981161
	*Metarhizium anisopliae*	0.002430735173
	*Trichosporon mucoides*	0.009765045696
Red Meat	*Bacteroides eggerthii*	0.065/−0.31
	*Staphylococcus epidermidis*	0.070/−0.31
	*Ruminococcus bromii*	0.077/0.30
	*Citrobacter gillenii*	0.030/−0.37
Fish	*Eubacterium dolichum*	0.037/−0.358
	*Cronobacter sakazakii*	0.087/−0.289
	*Haemophilus parainfluenzae*	0.072/−0.311
Vegetables	*Roseburia faecis*	0.096/−0.28
	*Faecalibacterium prausnitzii*	0.080/−0.304
	*Ruminococcus bromii*	0.066/−0.318
	*Veillonella parvula*	0.002/−0.496
	*Candida albicans*	0.0028/−0.496
Sweets	*Bifidobacterium adolescentis*	0.056/0.329
	*Blautia producta*	0.060/0.329
	*Agrobacterium sullae*	0.078/0.306

## Data Availability

The data presented in this study are available on request from the corresponding author.

## References

[1] Pearse A.G., Polak J.M. (1971). Neural crest origin of the endocrine polypeptide (APUD) cells of the gastrointestinal tract and pancreas. Gut.

[2] Dasari A., Shen C., Halperin D., Zhao B., Zhou S., Xu Y., Shih T., Yao J.C. (2017). Trends in the Incidence, Prevalence, and Survival Outcomes in Patients with Neuroendocrine Tumors in the United States. JAMA Oncol..

[3] Rindi G., Klimstra D.S., Abedi-Ardekani B., Asa S.L., Bosman F.T., Brambilla E., Busam K.J., de Krijger R.R., Dietel M., El-Naggar A.K. (2018). A common classification framework for neuroendocrine neoplasms: An International Agency for Research on Cancer (IARC) and World Health Organization (WHO) expert consensus proposal. Mod. Pathol..

[4] Sorbye H., Baudin E., Borbath I., Caplin M., Chen J., Cwikla J.B., Frilling A., Grossman A., Kaltsas G., Scarpa A. (2019). Unmet Needs in High-Grade Gastroenteropancreatic Neuroendocrine Neoplasms (WHO G3). Neuroendocrinology.

[5] Cénit M.C., Matzaraki V., Tigchelaar E.F., Zhernakova A. (2014). Rapidly expanding knowledge on the role of the gut microbiome in health and disease. Biochim. Biophys. Acta.

[6] Clemente J.C., Ursell L.K., Parfrey L.W., Knight R. (2012). The impact of the gut microbiota on human health: An integrative view. Cell.

[7] Carding S., Verbeke K., Vipond D.T., Corfe B.M., Owen L.J. (2015). Dysbiosis of the gut microbiota in disease. Microb. Ecol. Health Dis..

[8] DeGruttola A.K., Low D., Mizoguchi A., Mizoguchi E. (2016). Current Understanding of Dysbiosis in Disease in Human and Animal Models. Inflamm. Bowel Dis..

[9] Kho Z.Y., Lal S.K. (2018). The Human Gut Microbiome—A Potential Controller of Wellness and Disease. Front. Microbiol..

[10] Gopalakrishnan V., Helmink B.A., Spencer C.N., Reuben A., Wargo J.A. (2018). The Influence of the Gut Microbiome on Cancer, Immunity, and Cancer Immunotherapy. Cancer Cell.

[11] Shui L., Yang X., Li J., Yi C., Sun Q., Zhu H. (2020). Gut Microbiome as a Potential Factor for Modulating Resistance to Cancer Immunotherapy. Front. Immunol..

[12] White T.J., Bruns T., Lee S., Taylor J.W., Innis M.A., Gelfand D.H., Sninsky J.J., White T.J. (1990). Amplification and direct sequencing of fungal ribosomal RNA genes for phylogenetics. PCR Protocols: A Guide to Methods and Applications.

[13] Human Microbiome Project Consortium (2012). Structure, function and diversity of the healthy human microbiome. Nature.

[14] Zhang Y., Liang X.F., He S., Chen X., Wang J., Li J., Zhu Q., Zhang Z., Li L., Alam M.S. (2020). Effects of High Carbohydrate Diet-Modulated Microbiota on Gut Health in Chinese Perch. Front. Microbiol..

[15] Seo Y.S., Lee H.B., Kim Y., Park H.Y. (2020). Dietary Carbohydrate Constituents Related to Gut Dysbiosis and Health. Microorganisms.

[16] Fan X., Jin Y., Chen G., Ma X., Zhang L. (2021). Gut Microbiota Dysbiosis Drives the Development of Colorectal Cancer. Digestion.

[17] Vimal J., Himal I., Kannan S. (2020). Role of microbial dysbiosis in carcinogenesis & cancer therapies. Indian J. Med. Res..

[18] Li J.J., Zhu M., Kashyap P.C., Chia N., Tran N.H., McWilliams R.R., Bekaii-Saab T.S., Ma W.W. (2021). The role of microbiome in pancreatic cancer. Cancer Metastasis. Rev..

[19] Chattopadhyay I., Dhar R., Pethusamy K., Seethy A., Srivastava T., Sah R., Sharma J., Karmakar S. (2021). Exploring the Role of Gut Microbiome in Colon Cancer. Appl. Biochem. Biotechnol..

[20] Gupta H., Youn G.S., Shin M.J., Suk K.T. (2019). Role of Gut Microbiota in Hepatocarcinogenesis. Microorganisms.

[21] Nash A.K., Auchtung T.A., Wong M.C., Smith D.P., Gesell J.R., Ross M.C., Stewart C.J., Metcalf G.A., Muzny D.M., Gibbs R.A. (2017). The gut mycobiome of the Human Microbiome Project healthy cohort. Microbiome.

[22] Bruno G., Zaccari P., Rocco G., Scalese G., Panetta C., Porowska B., Pontone S., Severi C. (2019). Proton pump inhibitors and dysbiosis: Current knowledge and aspects to be clarified. World J. Gastroenterol..

[23] Vallianou N., Kounatidis D., Christodoulatos G.S., Panagopoulos F., Karampela I., Dalamaga M. (2021). Mycobiome and Cancer: What Is the Evidence?. Cancers.

[24] Blagojevic M., Camilli G., Maxson M., Hube B., Moyes D.L., Richardson J.P., Naglik J.R. (2021). Candidalysin triggers epithelial cellular stresses that induce necrotic death. Cell Microbiol..

[25] Haghi F., Goli E., Mirzaei B., Zeighami H. (2019). The association between fecal enterotoxigenic B. fragilis with colorectal cancer. BMC Cancer.

[26] Zamani S., Taslimi R., Sarabi A., Jasemi S., Sechi L.A., Feizabadi M.M. (2020). Enterotoxigenic Bacteroides fragilis: A Possible Etiological Candidate for Bacterially-Induced Colorectal Precancerous and Cancerous Lesions. Front. Cell Infect. Microbiol..

[27] Cheng W.T., Kantilal H.K., Davamani F. (2020). The Mechanism of Bacteroides fragilis Toxin Contributes to Colon Cancer Formation. Malays. J. Med. Sci..

[28] Boleij A., Hechenbleikner E.M., Goodwin A.C., Badani R., Stein E.M., Lazarev M.G., Ellis B., Carroll K.C., Albesiano E., Wick E.C. (2015). The Bacteroides fragilis toxin gene is prevalent in the colon mucosa of colorectal cancer patients. Clin. Infect. Dis..

[29] Viljoen K.S., Dakshinamurthy A., Goldberg P., Blackburn J.M. (2015). Quantitative profiling of colorectal cancer-associated bacteria reveals associations between fusobacterium spp., enterotoxigenic Bacteroides fragilis (ETBF) and clinicopathological features of colorectal cancer. PLoS ONE.

[30] Yurdakul D., Yazgan-Karataş A., Şahin F. (2015). Enterobacter Strains Might Promote Colon Cancer. Curr. Microbiol..

[31] Kaźmierczak-Siedlecka K., Roviello G., Catalano M., Polom K. (2021). Gut Microbiota Modulation in the Context of Immune-Related Aspects of Lactobacillus spp. and Bifidobacterium spp. in Gastrointestinal Cancers. Nutrients.

[32] Gao R., Kong C., Li H., Huang L., Qu X., Qin N., Qin H. (2017). Dysbiosis signature of mycobiota in colon polyp and colorectal cancer. Eur. J. Clin. Microbiol. Infect. Dis..

[33] Urbaniak C., Gloor G.B., Brackstone M., Scott L., Tangney M., Reid G. (2016). The Microbiota of Breast Tissue and Its Association with Breast Cancer. Appl. Environ. Microbiol..

[34] Wang Y., Wan X., Wu X., Zhang C., Liu J., Hou S. (2021). Eubacterium rectale contributes to colorectal cancer initiation via promoting colitis. Gut Pathog..

[35] Bhatt A.P., Redinbo M.R., Bultman S.J. (2017). The role of the microbiome in cancer development and therapy. CA Cancer J. Clin..

[36] Kennel K.B., Greten F.R. (2021). Immune cell—Produced ROS and their impact on tumor growth and metastasis. Redox Biol..

[37] Gagnière J., Raisch J., Veziant J., Barnich N., Bonnet R., Buc E., Bringer M.A., Pezet D., Bonnet M. (2016). Gut microbiota imbalance and colorectal cancer. World J. Gastroenterol..

[38] Lucas C., Barnich N., Nguyen H.T.T. (2017). Microbiota, Inflammation and Colorectal Cancer. Int. J. Mol. Sci..

[39] Vitale G., Dicitore A., Barrea L., Sbardella E., Razzore P., Campione S., Faggiano A., Colao A., Albertelli M., Altieri B. (2021). From microbiota toward gastro-enteropancreatic neuroendocrine neoplasms: Are we on the highway to hell?. Rev. Endocr. Metab. Disord..

[40] Strowski M.Z., Cramer T., Schäfer G., Jüttner S., Walduck A., Schipani E., Kemmner W., Wessler S., Wunder C., Weber M. (2004). Helicobacter pylori stimulates host vascular endothelial growth factor-A (vegf-A) gene expression via MEK/ERK-dependent activation of Sp1 and Sp3. FASEB J..

